# Influenza Virus Transmission from Horses to Dogs, Australia

**DOI:** 10.3201/eid1604.091489

**Published:** 2010-04

**Authors:** Peter D. Kirkland, Deborah S. Finlaison, Ellie Crispe, Aeron C. Hurt

**Affiliations:** Elizabeth Macarthur Agricultural Institute, Menangle, New South Wales, Australia (P.D. Kirkland, D.S. Finlaison); Warwick Farm Equine Centre, Warwick Farm, New South Wales, Australia (E. Crispe); World Health Organization Collaborating Centre for Reference and Research on Influenza, North Melbourne, Victoria, Australia (A.C. Hurt)

**Keywords:** Canine influenza, equine influenza, dogs, horses, respiratory disease, influenza, viruses, dispatch

## Abstract

During the 2007 equine influenza outbreak in Australia, respiratory disease in dogs in close contact with infected horses was noted; influenza (H3N8) virus infection was confirmed. Nucleotide sequence of the virus from dogs was identical to that from horses. No evidence of dog-to-dog transmission or virus persistence in dogs was found.

Respiratory disease in dogs caused by type A influenza virus was first noted in racing greyhounds in Florida in January 2004 (*1*). This subtype H3N8 virus has a presumptive but unidentified equine origin. The geographic extent of infection in racing greyhounds and in pet dogs suggest that this virus has become enzootic to the United States (*1*,*2*).

In the United Kingdom, pneumonia in dogs and influenza (H3N8) virus have been retrospectively linked, and subtype H3N8 infections have been identified serologically in dogs likely to have been in close contact with horses during the 2003 outbreak of equine influenza (*3*,*4*). A 78-bp segment of the hemagglutinin (HA) gene identified in dogs with pneumonia had complete homology with local equine strains (*3*). Unlike the situation in the United States, no evidence of continuing circulation of an influenza virus of equine origin in the canine population has been found in the United Kingdom.

In Australia, in late 2007, an outbreak of equine influenza virus (EIV) infection occurred in horses. During this outbreak, respiratory disease was noted in dogs of various ages and breeds that were kept near infected horses. Investigations were undertaken to exclude influenza virus infection.

## The Study

The first reported case was in a dog near a large stable; the dog became inappetant and lethargic and had had a slight nasal discharge and a persistent cough for several days. Over the next 2–3 weeks, dogs in or near stables with infected horses, including dogs whose owners were handling infected horses or dogs (n = 6) that were only housed with infected dogs, were examined. Samples were also collected from dogs kept with horses at 5 other locations 20–60 km from the first case. Of the 40 dogs, examined, 10 had clinical signs consistent with influenza (anorexia, lethargy, and, for some, a harsh cough that persisted for several weeks). All affected dogs recovered.

Nasal swabs and serum were collected from each of the 40 dogs; 23 were seropositive according to influenza type A blocking ELISA (*5*) and hemagglutinin inhibition (HI) assay (*5*) using A/equine/Sydney/2007 virus as antigen (Table). HI titers were 16–256 (geometric mean 122). Results were discordant for 5 dogs: for 2, HI titer was high but ELISA results were negative; for 3, ELISA results were positive but HI titer was negative. These discrepancies may have been resolved had later sampling been possible. Convalescent-phase serum samples were collected 14–16 days later from 26 of the dogs; seroconversion was noted for 4 of the 5 dogs with discordant ELISA and HI results. Testing of 19 dogs 2 years later showed no change in HI titer, although ELISA results were negative for each. Each seropositive dog had been in close proximity to EIV-infected horses but not always in direct contact. No evidence of lateral transmission was found for dogs that did not have contact with horses.

**Table:** Clinical signs and serologic findings for 40 dogs exposed to equine influenza virus, Australia, October 2007*

Dog	Breed	Age/sex	Clinical signs	Day of sample collection		PCR	Titer						
							ELISA				HI assay		
				1st	2nd		1st	2nd	3rd†		1st	2nd	3rd†
1	Cattle dog x	6 mo/F	Cough	5	NS	Neg	70	NA	NA		64	NA	NA
2	Whippet	6 mo/F	Cough, inappetance, lethargy, nasal discharge	5	25	Neg	79	69	Neg		128	128	128
3	Rottweiler	UK	Cough, lethargy	10	25	Neg	Neg	65	Neg		Neg	Neg	Neg
4	Dalmation	3 y/MN	Cough, inappetance, lethargy	11	NS	Neg	74	NA	NA		64	NA	NA
5	Kelpi x	9.5 y/FN	Cough, lethargy	12	26	Neg	66	74	NA		64	128	NA
6	Border collie	5 y/MN	Inappetance, lethargy	13	27	Neg	60	75	Neg		64	32	64
7	Cattle dog	4.5 y/M	Cough, lethargy	13	27	Neg	64	57	Neg		256	128	128
8	German shepherd	9 y/M	Inappetance, lethargy	14	30	Neg	Neg	Neg	NA		Neg	Neg	NA
9	Jack Russell	9 y/MN	Cough	14	30	Neg	Neg	65	NA		Neg	Neg	NA
10	Lowchen	2 y/MN	Cough, inappetance, lethargy, nasal discharge	26	NS	Neg	Neg	NA	Neg		Neg	NA	Neg
11	Cattle dog x	18 mo/F	None	10	26	Neg	77	75	NA		64	64	NA
12	Fox terrier x poodle	15 y/FN	None; lived with dog 4	11	NS	Neg	Neg	Neg	NA		Neg	Neg	NA
13	Kelpie x	4 y/MN	None	12	26	Neg	64	71	Neg		128	64	128
14	German shepherd	1.5 y/MN	None	12	26	Neg	71	67	Neg		128	64	64
15	Kelpie x labrador	10 y/FN	None	12	26	Neg	71	80	Neg		256	32	Neg
16	Cattle x kelpie	3 y/MN	None	12	27	Neg	64	61	Neg		128	32	64
17	Unknown	UK	None	12	27	Neg	66	66	Neg		256	64	32
18	Border collie	1 y/M	None	12	27	Neg	78	59	Neg		128	64	128
19	Jack Russell x	2.5 y/MN	None	12	26	Neg	49	71	NA		32	16	NA
20	Greyhound	18 mo/F	None	12	26	Neg	Neg	Neg	NA		256	128	NA
21	Greyhound	2 y. M	None	12	26	Neg	Neg	55	NA		Neg	Neg	NA
22	Greyhound	5 y/M	None	12	25	Neg	Neg	Neg	NA		Neg	Neg	NA
23	Greyhound	18 mo/F	None	12	25	Neg	Neg	Neg	NA		Neg	Neg	NA
24	Greyhound	18 m/F	None	12	25	Neg	Neg	Neg	NA		Neg	Neg	NA
25	Cattle x kelpie	4 y/FN	None	12	26	Neg	51	73	Neg		128	64	64
26	Poodle	5 mo/M	None, lived with dog 7	13	27	Neg	Neg	Neg	Neg		8	Neg	Neg
27	Jack Russell	5 y/F	None, lived with dog 5	13	27	Neg	Neg	Neg	NA		Neg	Neg	NA
28	Cattle x hunterway	4.5 y/M	None	14	30	Pos	Neg	50	NA		Neg	64	NA
29	Border collie	4 y/FN	None	15	NS	Neg	Neg	NA	NA		Neg	NA	NA
30	Cattle	13 y/MN	None	15	NS	Neg	Neg	NA	NA		>32	NA	NA
31	Jack Russell	UK	None	15	NS	Neg	76	NA	NA		64	NA	NA
32	Rottweiler	UK	None	15	NS	Neg	76	NA	Inc		128	NA	128
33	Fox terrier	4 y/MN	None	15	NS	Neg	78	NA	NA		32	NA	NA
34	Labrador	13 y/FN	None	15	NS	Neg	51	NA	NA		64	NA	NA
35	x	UK	None	18	NS	Neg	Neg	NA	Neg		Neg	NA	Neg
36	x	UK	None	18	NS	Neg	73	NA	Neg		64	NA	64
37	x	UK	None	18	NS	Neg	Neg	NA	Neg		Neg	NA	Neg
38	x	UK	None	18	NS	Neg	Neg	NA	NA		Neg	NA	NA
39	x	UK	None	18	NS	Neg	67	NA	Neg		256	NA	128
40	Poodle x	9 y/UK	None, lived with dog 10	26	NS	Neg	Neg	NA	Neg		Neg	NA	Neg

*HI, hemagglutination inhibition; x, cross-breed; NS, not sampled; neg, negative; NA, not applicable; pos, positive; UK, unknown; MN, male neutered; FN, female neutered. †Samples collected ≈2 y after second sample.

Nasal swabs from 1 clinically healthy dog had a positive result in an influenza A real-time reverse transcription–PCR assay (*5*) on 2 consecutive days. The dog remained clinically healthy and was seropositive (titer 64) on day 16 after the first positive swab was collected. Attempts to isolate virus from these swabs were unsuccessful.

Nucleic acid sequencing was conducted for the HA, neuraminidase (NA), and matrix (M) genes amplified by PCR from the RNA purified from 2 samples from this dog (A/canine/Sydney/6525/2007 and A/canine/Sydney/6692/2007) and from a nasal swab from an infected horse (A/equine/Sydney/6085/2007) in the same stable (GenBank accession nos. GU045761–GU045769). Sequences were aligned with representative sequences from GenBank by using Clustal W (www.clustal.org/) before phylogenetic trees with bootstrapping were generated (n = 1,000; random seed n = 111) with MegAlign (Lasergene; DNAStar, Madison, WI, USA). Complete nucleotide homology was found for each of the HA, NA, and M gene sequences from the 2 dogs and the sequence from the infected horse in the same stable (A/equine/Sydney/6085/2007).

When influenza subtype H3N8 sequences from horses and dogs were compared with other subtype H3N8 sequences in GenBank, the HA, NA, and M sequences were most similar to strains A/equine/Kanazawa/1/2007 and A/equine/Ibaraki/1/2007, which were isolated during the 2007 equine influenza outbreak in Japan (Figure). The HA, NA, and M gene sequences from the dogs in Australia were positioned on separate clades of the phylogenetic trees, as opposed to those from subtype H3N8 viruses from dogs in the United States, which all grouped closely together (Figure).

**Figure:**
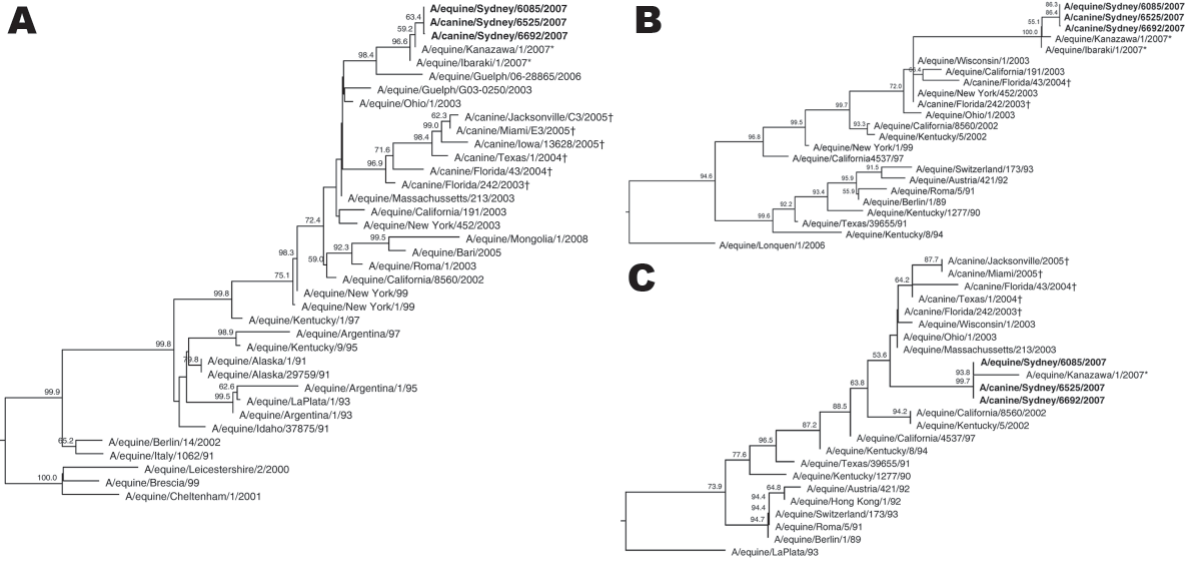
Phylogenetic trees of influenza subtype H3N8 viruses showing analyses conducted on A) hemagglutinin genes, B) neuraminidase genes, and C) matrix genes. Sequences from dogs are from the same animal on successive days. **Boldface** indicates viruses identified in dogs and horses in Australia, 2007, asterisks (*) indicate viruses from horses in Japan, and daggers (†) indicate viruses from dogs in the United States. Bootstrap values >50 are indicated at branch nodes. Bootstrap trials = 1,000; seed = 111.

## Conclusions

Researchers in Japan have described transmission of EIV from 3 experimentally infected horses to 3 dogs individually housed with each horse (*6*). Their findings were mostly consistent with ours, but there were some differences. Both studies showed direct linkage between active influenza virus infection in dogs and horses. Because some naturally infected dogs were only in the vicinity of stables and not in direct contact with horses, we believe that EIV may be readily transmitted from horses to dogs in close proximity. The mechanism of spread remains unclear, although in the United Kingdom aerosol transmission was believed to be a major means of spread to dogs (*4*). Studies conducted during the equine influenza outbreak in Australia indicate that the levels of virus excretion from horses not previously exposed to the virus can be extremely high (A.J. Read et al., unpub. data). Although humans readily spread virus from horse to horse, either directly during handling or by fomite transmission, human transmission of EIV to dogs that were not in the immediate vicinity of infected horses was not found. Similarly, dog-to-dog transmission was not found when infected dogs were transported and kept with other dogs in urban locations where there was no opportunity for contact with horses.

Although clinical signs were not observed for any of the dogs in Japan, >35% of the naturally infected dogs in Australia exhibited clinical signs, some quite severe and protracted. Nevertheless, virus was rarely detected in nasal secretions of the dogs in Australia, and there was no evidence of horizontal transmission to other dogs. The lack of clinical signs in experimentally infected dogs may be because of the small numbers of dogs or because of inoculum attenuation after passage in embryonated chicken eggs. That the experimentally infected dogs in Japan also had lower HI titers than did naturally infected dogs may be relevant.

Finally, when 19 of the dogs in Australia were tested 2 years after infection and without opportunity for reexposure, with only 1 exception, the HI antibody titers had not changed. This finding supports the interpretation that antibodies detected in dogs in the United Kingdom (*3*,*4*) had been acquired during the equine influenza outbreak several years earlier.

The nucleotide gene sequences encoding the 2 surface proteins (HA and NA) and the M protein from the infected dog in Australia matched those from the horse with which it had contact and did not have any of the nucleotide changes that have been identified in viruses from dogs in the United States (*2*). Such changes may be critical to, or a consequence of, the adaptation of EIVs to dogs and may play a role in enhancing the infectivity of these viruses for dogs because there is no evidence of continuing circulation of virus in dogs in Australia.

## References

[1] Crawford PC, Dubovi EJ, Castleman WL, Stephenson I, Gibbs EP, Chen L, Transmission of equine influenza virus to dogs. Science. 2005;310:482–5. 10.1126/science.1117950 16186182

[2] Payungporn S, Crawford PC, Kouo TS, Chen L, Pompey J, Castleman WL, Influenza A virus (H3N8) in dogs with respiratory disease, Florida. Emerg Infect Dis. 2008;14:902–8. 10.3201/eid1406.071270 18507900 PMC2600298

[3] Daly JM, Blunden AS, MacRae S, Miller J, Bowman SJ, Kolodziejek J, Transmission of equine influenza to English foxhounds. Emerg Infect Dis. 2008;14:461–4. 10.3201/eid1403.070643 18325262 PMC2570814

[4] Newton R, Cooke A, Elton D, Bryant N, Rash A, Bowman S, Canine influenza: cross-species transmission from horses. Vet Rec. 2007;161:142–3. 17660470 10.1136/vr.161.4.142-a

[5] Selleck PW, Kirkland PD. 2009. Avian influenza. In: Australian and New Zealand standard diagnostic procedures for animal diseases, Sub-Committee on Animal Health Laboratory Standards for Animal Health Committee, Australia [cited 2010 Feb 14]. http://www.scahls.org.au

[6] Yamanaka T, Nemoto M, Tsujimura K, Kondo T, Matsumura T. Interspecies transmission of equine influenza virus (H3N8) to dogs by close contact with experimentally infected horses. Vet Microbiol. 2009;139:351–5. 10.1016/j.vetmic.2009.06.015 19596528

